# Complexes of HMO1 with DNA: Structure and Affinity

**DOI:** 10.3390/biom14091184

**Published:** 2024-09-20

**Authors:** Daria K. Malinina, Grigoriy A. Armeev, Olga V. Geraskina, Anna N. Korovina, Vasily M. Studitsky, Alexey V. Feofanov

**Affiliations:** 1 Biology Faculty, Lomonosov Moscow State University, Moscow 119992, Russia; daria99malinina@gmail.com (D.K.M.); armeev@intbio.org (G.A.A.); olgsamsonova@yandex.ru (O.V.G.); anna.korovina@gmail.com (A.N.K.); vasily.studitsky@fccc.edu (V.M.S.); 2 Fox Chase Cancer Center, Philadelphia, PA 19111-2497, USA; 3 Shemyakin-Ovchinnikov Institute of Bioorganic Chemistry, Russian Academy of Sciences, Moscow 117997, Russia

**Keywords:** HMO1, high-mobility group B, DNA, protein DNA complex, molecular dynamics, EMSA, CD spectroscopy

## Abstract

*Saccharomyces cerevisiae* HMO1 is an architectural nuclear DNA-binding protein that stimulates the activity of some remodelers and regulates the transcription of ribosomal protein genes, often binding to a DNA motif called IFHL. However, the molecular mechanism dictating this sequence specificity is unclear. Our circular dichroism spectroscopy studies show that the HMO1:DNA complex forms without noticeable changes in the structure of DNA and HMO1. Molecular modeling/molecular dynamics studies of the DNA complex with HMO1 Box B reveal two extended sites at the N-termini of helices I and II of Box B that are involved in the formation of the complex and stabilize the DNA bend induced by intercalation of the F114 side chain between base pairs. A comparison of the affinities of HMO1 for 24 bp DNA fragments containing either randomized or IFHL sequences reveals a twofold increase in the stability of the complex in the latter case, which may explain the selectivity in the recognition of the IFHL-containing promoter regions.

## 1. Introduction

High-mobility group B (HMGB) factors are a family of highly abundant architectural nonhistone proteins, which participate in DNA transcription, repair, replication and recombination [1]. HMGB proteins serve as crucial modulators of chromatin structure, at least in part because their interaction with DNA is accompanied by DNA bending [2]. 

HMO1 from *Saccharomyces cerevisiae* is a member of the HMGB family. It is a nuclear protein that is essential for normal cell growth, the maintenance of plasmid stability and the regulation of chromatin sensitivity to nucleases [3]. HMO1 stimulates the activity of SWI/SNF (switching/sucrose nonfermenting), ISW1a (imitation switch complex 1a) and RSC (remodeling the structure of chromatin) chromatin remodelers [4,5] and supports the unwinding of nucleosomes by FACT (facilitates chromatin transcription) [6]. HMO1 acts as an architectural protein: it strongly bends DNA [7] and reorganizes nucleosomes [6,8]. HMO1 regulates the positioning of nucleosomes on the promoters of ribosome protein genes (RPGs) and maintains nucleosome-free regions at actively transcribed rRNA genes [9]. HMO1 is actively involved in transcription by RNA polymerases I and II [10,11,12].

HMO1 is classified as a non-sequence-specific DNA-binding protein in vitro [13,14]. However, HMO1 is associated with about half of all RPG promoters in vivo [15], and this binding highly correlates with the localization of the specific DNA motif called IFHL [10]. The presence of the IFHL motif is not sufficient for HMO1 association with RPGs but is supposed to contribute to the binding of HMO1 to RPG promoters [10,14,15,16,17].

**Figure 1:**
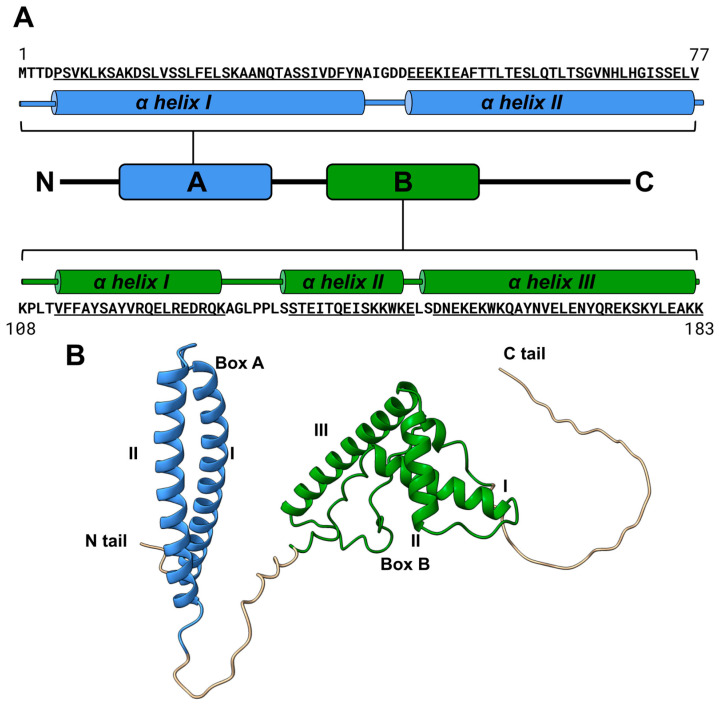
Structure of *S. cerevisiae* HMO1 protein. (**A**) Amino acid sequence and the domain structure of HMO1. (**B**) Structure of HMO1 predicted by AlphaFold2 (https://alphafold.ebi.ac.uk/entry/Q03973 model built 1 November 2022). Both Box A and Box B domain models have high AlphaFold confidence scores (>90%, with the occasional decrease to 70–90%). Linker and C-tail fragments have low confidence scores.

HMO1 has two DNA-binding domains (HMG boxes designated as Box A and Box B) connected by a linker and a C-terminal fragment containing an extended region of basic amino acids (Figure 1) [3]. No high-resolution structures of either full-length HMO1 or its individual domains have been solved. According to the prediction made using AlphaFold2 (https://alphafold.ebi.ac.uk/entry/Q03973, accessed on 6 October 2023), Box A comprises two α-helices (this structure is unusual for HMG boxes), while Box B forms three α-helices that are typical for the HMG box L-shaped fold. Linker and C-tail fragments are characterized by very low confidence scores in this model, suggesting that they are intrinsically disordered or non-structured [18].

Both HMG boxes of HMO1 are essential for DNA binding, but their affinities and DNA-bending abilities are different [14]. Box A possesses a lower affinity to DNA but a better DNA-bending ability compared to Box B [13]. The C-terminal region does not affect the HMO1 affinity to DNA but is essential for introducing a bend into DNA [19,20].

Currently, many aspects of the formation of HMO1 complexes with DNA are still unknown, including the issue of how strongly the DNA sequence, in particular, the IFHL sequence, affects HMO1 affinity.

In this work, the structural changes in HMO1 and DNA during complex formation were analyzed using circular dichroism (CD) and fluorescent spectroscopy. The structure of the complex between Box B of HMO1 and DNA was characterized using homology-based molecular modeling and molecular dynamics (MD) simulations. HMO1 interactions with DNA containing either randomized or IFHL sequences were studied using an electrophoretic mobility shift assay (EMSA), and the dissociation constants of the complexes were measured.

## 2. Materials and Methods

### 2.1. HMO1 Expression and Purification

The HMO1 coding sequence was amplified from the genomic DNA of *Saccharomyces cerevisiae* by polymerase chain reaction (PCR) using the following primers modified to introduce NdeI and XhoI sites at ends of the PCR product: forward primer 5′-TATAACATATGACTACAGATCCTTCTG-3′ (the NdeI site is underlined) and reverse primer 5′-GTAATCTCGAGTCAAATAGAAGAGTTGGATTTGT-3′ (the XhoI site is underlined). The PCR fragment was digested with NdeI and XhoI restriction endonucleases (New England Biolabs, Ipswich, MA, USA) and then inserted into the modified plasmid pET15b to generate a plasmid pET15b-HMO1-expressing N-terminally His (x6)-tagged HMO1 (wild-type) in *E. coli* cells.

Rosetta 2(DE3) cells (Novagen, Merck KGaA, Darmstadt, Germany) transformed with pET15b-HMO1 were grown in 1 L of lysogeny broth medium containing 100 µg/mL of ampicillin at 37 °C to an optical density of 0.6–0.8 optical units per cm at a 600 nm wavelength, induced with 0.5 mM of isopropyl β-D-1-thiogalactopyranoside for 3 h at 30 °C and harvested by centrifugation (3200× *g*) for 30 min. Cells were resuspended in 50 mL of phosphate-buffered saline, sedimented again and frozen at −80 °C.

After thawing, the cells were resuspended in a lysis buffer (50 mM of Tris-HCl at pH 8.0, 0.5 M of NaCl, 4 mM of β-mercaptoethanol, 1 mM of EDTA, 10% glycerol, 0.1% Triton X-100, 1 mM of phenylmethylsulphonyl fluoride and complete-EDTA-free protease inhibitor tablets (Roche, Basel, Switzerland)) and disrupted by sonication on ice using a Sonic Dismembrator FB120 (Thermo Fisher Scientific, Waltham, MA, USA).

The cell lysate was cleared by centrifugation (11,000× *g*, 30 min, 4 °C). The supernatant was filtered through a syringe filter with a pore diameter of 0.2 μm and passed through a His-tag protein purification column (HisTrap HP, GE Healthcare, Chicago, IL, USA) pre-equilibrated with the lysis buffer. The protein was washed sequentially with the lysis buffer and wash buffer (a lysis buffer containing 10 mM of imidazole) and eluted with an elution buffer (50 mM of Tris-HCl at pH 8.0, 0.3 M of NaCl, 4 mM of β-mercaptoethanol, 1 mM of EDTA, 10% glycerol, 250 mM of imidazole, 1 mM of phenylmethylsulphonyl fluoride and 0.1% Triton X-100). The resulting protein fraction was diluted twice using a null buffer (50 mM of Tris-HCl at pH 8.0, 4 mM of β-mercaptoethanol, 1 mM of EDTA and 10% glycerol) and further purified by a cation exchange chromatography column (HiTrap Heparin, GE Healthcare) equilibrated with a HepA buffer (50 mM of Tris-HCl at pH 7.5, 150 mM of NaCl, 10% glycerol and 4 mM of β-mercaptoethanol). The protein was eluted with a 0–100% salt gradient using a HepB buffer (50 mM of Tris-HCl at pH 7.5, 2 M of NaCl, 10% glycerol and 4 mM of β-mercaptoethanol). Fractions that contained the protein were collected, concentrated and passed through a Sephacryl gel filtration column (GE Healthcare) equilibrated with a sizing buffer (20 mM of HEPES-Na at pH 8.0, 0.3 M of NaCl, 0.1 mM of tris(2-carboxyethyl)phosphine and 5% glycerol). The protein was concentrated by centrifugation (5000× *g*, 30 min, 4 °C) in an Amicon^®^ Ultra-15 centrifugal filter unit (Millipore, Merck KGaA, Darmstadt, Germany).

### 2.2. Oligonucleotides

Oligonucleotides of 24 bp length were purchased from Lumiprobe (Moscow, Russia). For the RO duplex with a randomized sequence, the following oligonucleotides were used: 5′-[Cy3]CTATCGGCACACTTCACGGCACAC-3′ and 5′-GTGTGCCGTGAAGTGTGCCGATAG-3′, where Cy3 is the fluorescent label cyanine 3. For the SO duplex containing an IFHL site, the following oligonucleotides were used: 5′-ACTACGCTCTAGGCAGAGTTCACC-3′ and Cy3-labeled 5′-[Cy3]GGTGAACTCTGCCTAGAGCGTAGT-3′ (the IFHL site is underlined). To form dsDNA, pairs of oligonucleotides were mixed in equimolar concentrations (0.5–3 μM) in an annealing buffer (10 mM of Tris-HCl at pH 8.0, 50 mM of NaCl and 1 mM of EDTA), incubated for 5 min at 95 °C and slowly (~1 h) cooled at room temperature.

### 2.3. Formation of Complexes and Their Analysis with EMSA

Titration of RO or SO (6 nM) with increasing concentrations of HMO1 (10–800 nM) was performed in buffer A (17 mM of HEPES at pH 7.6, 2 mM of Tris-HCl, 0.8 mM of Na_3_EDTA, 0.11 mM of 2-mercaptoethanol, 11 mM of NaCl, 1.1% glycerol and 12% sucrose). The complexes were incubated for 30 min at 30 °C and subjected to analysis using an EMSA.

The EMSA was performed in the 8% polyacrylamide gel in 0.5× TBE buffer (45 mM of Tris-HCl, 45 mM of boric acid and 1 mM of EDTA) at 120 V for 40 min. The DNA and HMO1:DNA complexes were detected in gels using a Typhoon Trio imager (GE Healthcare) with excitation at 532 nm and emission at 580 nm (Cy3 fluorescence).

### 2.4. Gel Image Analysis

A densitometric analysis of the gel images was performed using the ImageJ software package, version 1.53k (NIH, Bethesda, MD, USA). The relative changes in the brightness of free DNA bands upon titration with HMO1 were used to calculate the extent of DNA binding to HMO1 (α) in percentages:

*α*(*C*) = (1 − (*I*(*C*)/*I_0_*)) × 100 (1)

where *I*(*C*) and *I*_0_ are the brightnesses of the DNA bands at the HMO1 concentration *C* and without HMO1, respectively.

The data of *α*(*C*) were fitted with the equation:

*α*(*C*) = 100 × *C^n^*^/^(*K_d_^n^* + *C^n^*) (2)

where *K_d_* is an apparent dissociation constant of the HMO1:DNA complexes, and *n* is the Hill coefficient.

*K_d_* values were determined in three independent experiments and averaged.

### 2.5. CD Spectroscopy

All the samples (36 µM of HMO1, 36 μM of dsDNA and the equimolar mixture of HMO1 and non-labeled DNA (36 µM each)) were prepared in a CD buffer (20 mM of HEPES at pH 8.0, 80 mM of NaCl, 1 mM of EDTA, 1% glycerol and 0.02 mM of tris(2-carboxyethyl)phosphine). Equimolar amounts (36 µM each) of HMO1 and non-labeled DNA were mixed and incubated on ice for 1 h.

CD spectra were measured using a Jasco-810 spectropolarimeter (Jasco, Tokyo, Japan) in the 190–330 nm range with a 0.2 nm scanning step, using a quartz cuvette with a 0.1 mm optical path length (Hellma Switzerland AG, Buchs, Switzerland). Each spectrum was averaged over four repeated scans. The experiments were carried out in two independent replicates. The spectra of HMO1 and the HMO1:DNA complexes were corrected for the baseline measured from the CD buffer and DNA in the CD buffer, respectively, and the protein structure was analyzed using CDPro software, version 1.3 with the SMP56 reference set of proteins [21].

### 2.6. Fluorescence Spectroscopy

Fluorescence spectra of HMO1 and the HMO1:DNA complexes were measured using a Cary Eclipse fluorescence spectrophotometer (Varian, Palo Alto, CA, USA) with a quartz ultra-microcell of 2 × 10 mm size (Hellma Switzerland AG, Switzerland). Fluorescence was excited at 280 nm and recorded in the 295–450 nm range with 2 nm spectral resolution. The baseline was measured from the buffer or a solution of oligonucleotides (150 nM) in the buffer and was subtracted from the spectra. To minimize the factors of fluorescence fading and protein adsorption at the cuvette over time, each spectrum was obtained from a new sample containing 150 nM of HMO1 and a particular concentration of DNA (10–200 nM), which was pre-incubated on ice for 1 h.

### 2.7. Homology Modeling

HMO1 Box B was taken from AlphaFoldDB (https://alphafold.ebi.ac.uk/entry/Q03973, time of access 6 October 2023). Then, we constructed a ternary structure based on the TFAM:DNA complex (PDB:6hb4 [22]) using the SwissModel web service [23]. We utilized this ternary structure to align the HMO1 Box B model with DNA. In other words, we combined the AlphaFold2 model (the protein section) with the DNA model from the TFAM:DNA complex (PDB:6hb4) using the match-align tool from UCSF Chimera [23]. Next, we replaced the DNA sequence and extended the DNA to match the RO sequence and positioned HMO1 Box B in contact with the minor groove. Finally, we optimized the DNA backbone using the Phenix optimize geometry tool [24].

### 2.8. MD and Trajectory Analysis

GROMACS 2020.1 [25] was utilized for MD simulations, employing the AMBER ff14SB [26] force field with the addition of parmbsc1 DNA and CUFIX [27] ion parameter corrections. The model systems were positioned (2 nm distance to the walls) within a periodic simulation box of a truncated octahedron shape. A TIP3P [28] water model was employed for solvation, and Na^+^ and Cl^−^ ions were introduced to maintain charge neutrality and achieve an ionic strength of 150 mM. Prior to the simulations, the models underwent a minimization process using the steepest-descent gradient method with 10,000 steps, followed by 5-step equilibration. The equilibration involved gradually reducing the restraining potential in the first four steps and then allowing unrestrained equilibration for 200 ps in the fifth step. Harmonic positional restraints with values of 500, 50, 5 and 0.5  kJ mol^−1^ nm^−2^ were used during the first 100 ps step and the next three steps of 200 ps each, respectively. The temperature was set to 300 °C, and the pressure was maintained at 1 bar. During the MD production runs, trajectory frames were outputted at a frequency of 1 ns with a 2 fs integration step. The simulations were run for a total of 1000 ns, both for free HMO1 Box B and HMO1 Box B with DNA.

Trajectory analysis was conducted using custom scripts written in Python 3 and the MDAnalysis [29] library. The initial model was used as a reference for aligning all trajectories using root-mean-square deviation (RMSD) with the C_α_ atoms of the protein (Figure S1A). Protein–DNA contacts were defined as pairs of non-hydrogen atoms within a distance of less than 4 Å. Contact profiles were calculated for each frame and then averaged. Additionally, the solvent-accessible surface areas for tryptophan residues were calculated for each frame using the FreeSASA, version 2.1.2 [30] library and then averaged.

## 3. Results and Discussion

### 3.1. Production of HMO1 and Oligonucleotide Duplexes

**Figure 2:**
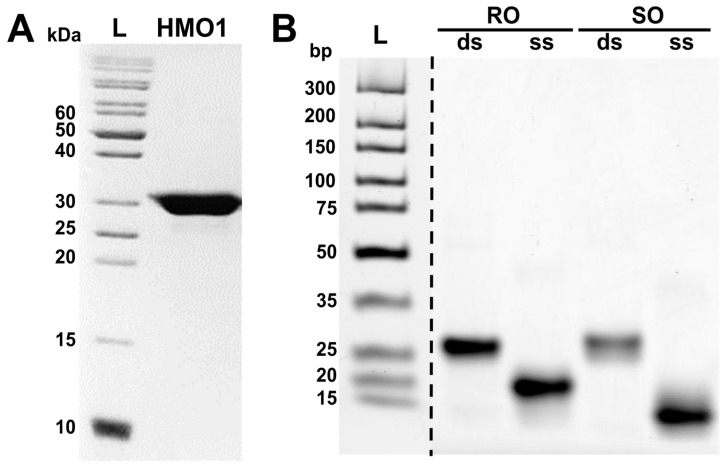
Gel electrophoresis of the purified HMO1 protein and annealed DNA duplexes used in the study. (**A**) SDS PAGE of the purified HMO1 protein. L—ladder of protein markers. (**B**) Native PAGE of RO and SO oligonucleotides. L—ladder of DNA markers, ds and ss—double-stranded and single-stranded DNA.

To study the properties of HMO1:DNA complexes, His6-Hmo1 protein was expressed in *E. coli* Rosetta 2(DE3) cells using a constructed pET-15b-HMO1 plasmid. The highest protein production was achieved when induction with isopropyl β-D-1-thiogalactopyranoside was performed for 3 h at 30 °C. Recombinant HMO1 protein was obtained by sequential purification using affinity, cation exchange and gel-exclusion chromatography (Figure 2A). The yield of HMO1 was 3 mg per 1 L of *E. coli* culture.

To investigate whether the affinity of HMO1 to DNA depends on the DNA sequence, we constructed two 24 bp DNA duplexes: RO and SO. RO contained a randomized sequence, while SO contained the IFHL site sequence TCTAGGCAGAG. One of the DNA strands in these duplexes was labeled with the fluorescent label Cy3. Annealing of equimolar amounts of single-stranded oligonucleotides by heating to 95 °C for 5 min, followed by cooling of the sample at room temperature, led to the formation of duplexes without the impurities of single-stranded DNA (Figure 2B).

### 3.2. Structural Analysis of HMO1 and Its Complex with DNA

**Figure 3:**
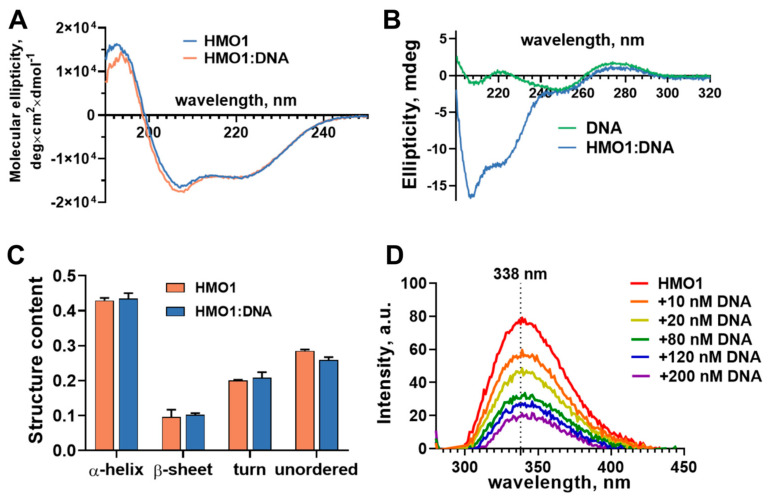
Spectral analysis of HMO1 and HMO1:DNA complexes. (**A**) CD spectra of HMO1 and the HMO1:DNA complex (the contribution of DNA was subtracted). (**B**) CD spectra of DNA and the HMO1:DNA complex in the region where the contribution of the DNA signal dominates. (**C**) Analysis of the secondary structure of HMO1 and HMO1:DNA complexes based on the CD spectra. (**D**) Changes in the fluorescence spectrum of the HMO1 protein induced by formation of the complex with DNA.

The secondary structure of HMO1 and its changes upon the formation of complexes with DNA (the RO duplex) were studied by CD spectroscopy (Figure 3A). Analysis of the CD spectrum of HMO1 shows that the α-helix and unordered structures dominate in the secondary structure of HMO1 (Figure 3C) in accordance with the earlier published data [13] and the AlphaFold2 prediction. The RO duplex has the CD spectrum with two positive (190–204 and 261–300 nm) and two negative (204–216 and 225–261 nm) bands, which are characteristics of the B form of DNA [31]. Analyzing the CD spectrum of the HMO1:RO complex, one should consider that the RO spectrum overlaps with that of HMO1 in the 190–240 nm region, but the contribution of the protein signal at wavelengths longer than 240 nm is negligible. In the 240–300 nm region, the spectrum of RO in the complex with HMO1 is very similar to the spectrum of protein-free RO (Figure 3B), thus leading to the conclusion that no significant changes occur in the RO structure during the complex formation.

The similarity of spectra in the 240–300 nm region allows for subtracting the contribution of the DNA signal from the spectrum of the HMO1:RO complex and using the residual spectrum for the analysis of the secondary structure of HMO1 in the complex with the RO duplex. This analysis shows that the secondary structure of HMO1 also does not change in the complex (Figure 3C).

The fluorescence spectrum of HMO1 (Figure 3D) is mainly due to the emission of two tryptophan residues (W149 and W160) localized within helix II of Box B. The spectrum has a maximum of 338 nm, which likely indicates the polar environment of the side chains of tryptophan residues [32]. The formation of HMO1:RO complexes is accompanied by the quenching of tryptophan fluorescence without changes in the maximum and shape of the spectrum (Figure 3D). Taking into account the absence of structural changes in HMO1 during complex formation with DNA, this likely indicates that one or both tryptophan residues are localized at the interaction interface.

To analyze the details of HMO1:DNA complex formation in the absence of the high-resolution structures of HMO1 and the HMO1:DNA complex, we employed the methods of homology-based molecular modeling and MD.

A search for the structures of DNA–protein complexes suitable for homology-based MD of the HMO1:DNA complex reveals several high-resolution structures of DNA complexes with human mitochondrial transcription factor A (TFAM), whose HMG1 domain has 30% homology with Box B of HMO1 and the lack of high-resolution structures of DNA complexes with proteins that contain domains homologous to Box A of HMO1. Accordingly, only the DNA complex with HMO1 Box B (HMO1BB) was modeled.

**Figure 4:**
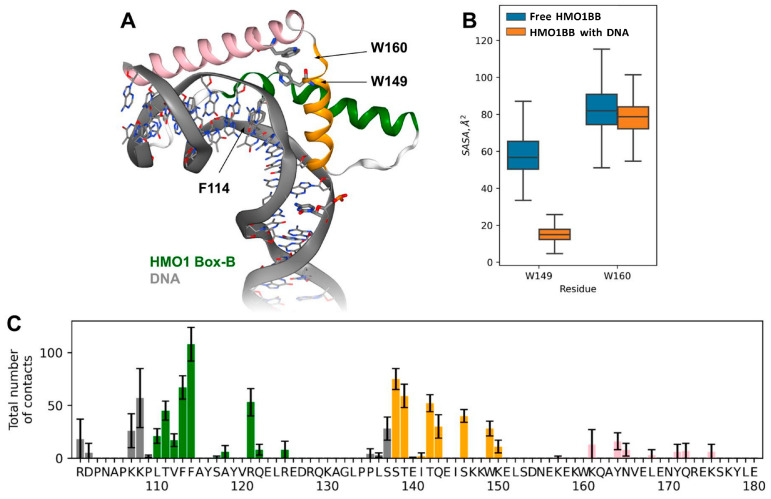
Modeling of the DNA complex with HMO1BB. (**A**) Homology-based model of the complex between DNA and HMO1BB. Alpha helices I, II and III are shown in green, yellow and pink, respectively. DNA backbone and unordered regions of HMO1BB are shown in gray. (**B**) Solvent-accessible surface areas of W149 and W160 residues of intact and DNA-bound HMO1BB calculated from MD trajectories. (**C**) HMO1BB contacts with DNA calculated from MD trajectories. Residues of α-helices I, II and III are shown in green, yellow and pink, respectively. Residues of unordered regions of HMO1BB are shown in gray.

A model of the HMO1BB complex with DNA was built using the three-stage hybrid modeling approach. First, the model of HMO1BB was created using AlphaFold2, as it provides more accurate results than single-template homology modeling. Second, the modeled HMO1BB structure was superimposed on the TFAM:DNA complex (PDB:6hb4) [22] using sequence-based structure alignment. Third, the DNA model was modified and extended to match the DNA (RO) length and sequence (Figure 4A). Both the model of free HMO1BB and the model of the HMO1BB:DNA complex were subjected to MD simulations.

During the 1000 ns of MD simulations, HMO1BB preserved its initial L-shaped fold and did not show any signs of structural instability. The RMSD fluctuated within the 1.5–3 Å range (Figure S1A,B). No significant internal mobility was observed.

Analysis of the resulting HMO1BB structure shows that it is maintained by interactions between the N-termini of helices I and II, which form the short branch of the L-fold, and between the C-terminus of helix III and the non-structured N-tail that form the long branch of the L-fold. The stability of the latter structure is mainly maintained by a network of long-living hydrogen bonds formed between Y164, Y171 and Y178 of helix III and the non-structured N-tail protein backbone. The small helixes are tightly bound through ionic interactions between R128 and E144 and between D127 and K148. There are also hydrophobic contacts between F113, Y116, W149, L152 and W160 on the interfaces between three helices. Residues W149 and W160 are partially accessible to the solvent, which is consistent with the observed position of the maximum in the fluorescence spectrum of HMO1 (Figure 3D).

During the MD simulations, the HMO1BB:DNA complex maintained its overall shape and stability (Figure S1A,C). DNA is severely bent in the obtained model, and this HMO1BB-induced kink is caused by the intercalation of the F114 side chain between the base pairs of DNA (Figure 4A). The intercalation locally disrupts base pair stacking, resulting in a strong DNA bend, which is preserved throughout the entire MD simulation trajectory. Except for this local structure disturbance, essential parameters of the B form of DNA are preserved in the complex. F114 has hydrophobic contacts with closely positioned L110 and F113 residues; together, these three residues form a hydrophobic/intercalation (H/I) wedge [33,34].

The HMO1BB complex with bent DNA is stabilized by the interactions of N-terminal parts of helices I and II with a minor DNA groove. These interactions are consistently preserved during the MD simulations (Figure 4C and Figure S1D). The positively charged amino acids K107, K108, and R121 (helix I), along with the polar amino acids (S138, S139, S146) of helix II and the nonpolar residues F113, F114 and W149, are involved in the formation of contacts with DNA. Contacts between helix III and DNA are very unstable and hardly contribute to the complex formation (Figure 4C).

The obtained model of the DNA:HMO1BB complex is consistent with CD data on the absence of changes in the structure of DNA and HMO1 during complex formation. It is also consistent with the fluorescent spectroscopy data on the localization of tryptophan residue at the interaction interface. It is W149 in HMO1BB that interacts with DNA forming contacts with the sugar–phosphate backbone. The solvent-accessible surface area of W149 is significantly decreased in the complex in contrast to that of W160 (Figure 4B), which is not directly involved in complex formation. W149 is conserved within the HMGB protein family and is typically positioned at the protein–DNA interface [35,36,37,38,39,40,41].

A comparison of the affinities of HMO1 to 24 bp dsDNA containing either randomized or IFHL site sequences reveals the sequence specificity of HMO1 binding, which likely facilitates recognition of the IFHL-containing promoter regions of ribosome protein genes. In general, the mode of HMO1BB interaction with DNA is similar to that reported for HMGB proteins, which induces the DNA bend due to intercalation of a single H/I wedge localized in α-helix I [38,42,43]. In most cases, the intercalating residue is I, M or F, as in HMO1B. Another group of HMGB proteins has the second intercalating wedge at α-helix II that is formed by one or two nonpolar amino acids [40]. In the homological region, HMO1BB has a polar amino acid, S138, which cannot intercalate into DNA.

The ability of HMGB proteins to bind DNA in a sequence-specific or non-sequence-specific manner was proposed to be partially based on the number of intercalating wedges: one or two, respectively [33,40,41,44]. Accordingly, HMO1BB is expected to provide sequence-specificity to the HMO1 protein. At the same time, HMO1BB does not have another determinant of sequence-specificity, asparagine residue at position 111. HMO1BB has T111, which is structurally more similar to serine, the signature of non-sequence-specific proteins. Thus, HMO1BB contains determinants of both sequence-specific and non-sequence-specific HMGB proteins.

### 3.3. Affinity of HMO1 to DNA

**Figure 5:**
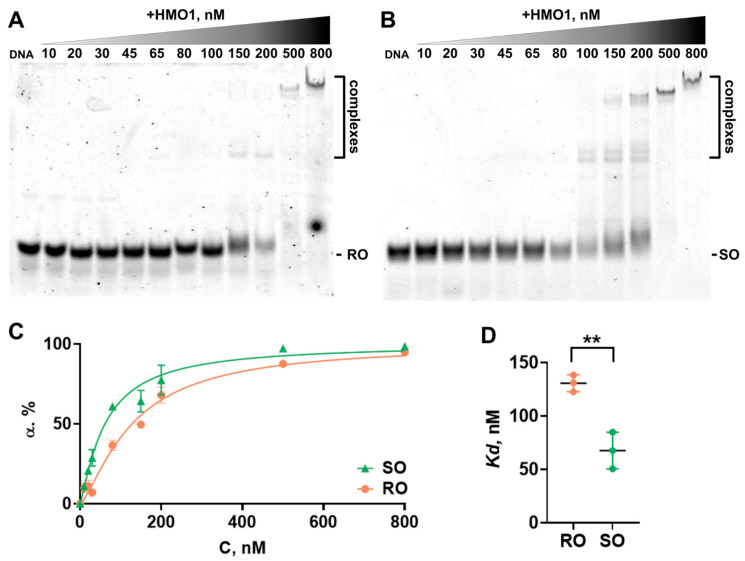
Affinity of HMO1 to DNA. (**A**,**B**) Electrophoresis of HMO1 complexes with RO (**A**) and SO (**B**) oligonucleotides containing either randomized or IFHL site sequences, respectively. (**C**) Isotherms of HMO1 binding to RO and SO oligonucleotides calculated using the electrophoresis data on the changes in the amount of free DNA. Data (mean ± SEM) were averaged over three independent experiments. α—the extent of DNA binding to HMO1, C—HMO1 concentration. (**D**) Comparison of *K_d_* values of HMO1 complexes with RO and SO (mean ± SEM, n = 3). ** *p* = 0.005 according to the *t*-test. Test for normality (Shapiro–Wilk): W = 0.99, *p* = 0.98.

To study whether HMO1 binds to DNA in a sequence-specific manner, the formation of the HMO1:DNA complex was analyzed using an EMSA. The addition of HMO1 to the RO duplex (6 nM) resulted in complex formation at 100–200 nM HMO1 (Figure 5A,C). When most of the RO duplex was already in the complex, a further increase in the HMO1 concentration to 500–800 nM resulted in the formation of a new complex with very low mobility in the gel (Figure 5A). Considering the prominent changes in the mobility and the reported tendency of HMO1 to self-association, this low-mobility HMO1:RO complex likely contains two or more HMO1 molecules. As was shown earlier, self-associates of HMO1 contain four or more molecules of the protein [20,45].

According to the analyses of the decrease in free DNA content in the gel (Figure 5C), the *K_d_* of the HMO1:RO complex is 128 ± 15 nM (Figure 5D).

Complex formation between HMO1 and the SO duplex (6 nM) was observed at 65–200 nM HMO1 (Figure 5B,C) and was accompanied by the formation of two types of complexes with slightly different mobilities in the gel (Figure 5B). These complexes probably correspond to two modes of interactions of the SO duplex with Box A and Box B of HMO1. The formation of these complexes occurs at a lower concentration of HMO1 compared to the RO duplex. The formation of the additional low-mobility complex, which can be assigned to the HMO1:SO complex containing several HMO1 molecules, was observed at 150–800 nM HMO1 (Figure 5B).

The presence of the IFHL site in DNA leads to a statistically significant increase in the affinity of HMO1 to DNA. The *K_d_* of the HMO1:SO complexes is 62 ± 8 nM (Figure 5D).

Previously, the length of the HMO1 binding site at the DNA was estimated to be 26 bp [13]. According to our data, DNA with a length of 24 bp also forms stable complexes with HMO1. However, the *K_d_* values of the HMO1 complexes with 24 bp DNA are notably increased compared with the previously reported values of 40 ± 5 nM [13] and 26.5 ± 1.1 nM [19].

As shown *in vivo*, HMO1 binding to RPG promoters is Rap1-dependent and occurs predominantly at the sites that contain IFHL sequence(s) [10,15]. Increased HMO1 binding to promoters was observed if they contained several IFHL sites [15]. Even in the absence of Rap1 (repressor-activator protein 1), the binding of HMO1 to DNA was increased *in vitro* when DNA contained several IFHL sites [15]. Our data confirm a sequence-specific increase in HMO1 binding to DNA and show that in the case of the protein interaction with the IFHL site, the *K_d_* of the complex is reduced by two times compared to DNA with a randomized sequence.

Among other HMG proteins known as non-sequence-specific DNA binders, HMGD protein is known to have some selectivity to the A/T-rich sequences containing TG dinucleotide [46], while TFAM protein binds more strongly to specific promoter sequences compared to other sites of DNA [47]. Thus, HMO1 belongs to this group of non-sequence-specific DNA binders, which have an increased affinity to particular DNA sequences. 

## 4. Conclusions

Although HMO1 is classified as a non-sequence-specific DNA binding protein [14], our data show it has increased affinity to particular DNA sequences. One of such sequences is the IFHL motif, which is often present in RPG promoters. According to our data, the complex stability when HMO1 binds to the IFHL motif increases two times compared to a randomized DNA sequence. This contributes to the observed accumulation of HMO1 protein at RPG promoters, especially when they contain multiple IFHL motifs, and likely contributes to the fine regulation of transcriptional activation in yeast cells.

## Data Availability

The structures of HNO1BB and its complex with DNA, along with the MD trajectories, can be found on the Zenodo repository at https://zenodo.org/records/13759091. Other data presented in this study are available upon request from the corresponding author. The data are not publicly available due to local regulations.

## Supplementary Materials

The following supporting information can be downloaded at: https://www.mdpi.com/article/10.3390/biom14091184/s1, Figure S1. Modeling of the HMO1BB complex with DNA.
