# Supplementary material for: Complexes of HMO1 with DNA: Structure and Affinity
Source: Biomolecules. 2024 Sep 20;14(9):1184. doi: 10.3390/biom14091184 (PMC11430298; doi:10.3390/biom14091184)
Supplement: Supplementary file 1 [file biomolecules-14-01184-s001.zip › biomolecules-3179600-supplementary.pdf]

Supplementary materials to the manuscript

Complexes of HMO1 with DNA: structure and affinity

Daria K. Malinina, Grigoriy A. Armeev, Olga V. Geraskina, Anna N. Korovina, Vasily M. Studitsky, Alexey V. Feofanov

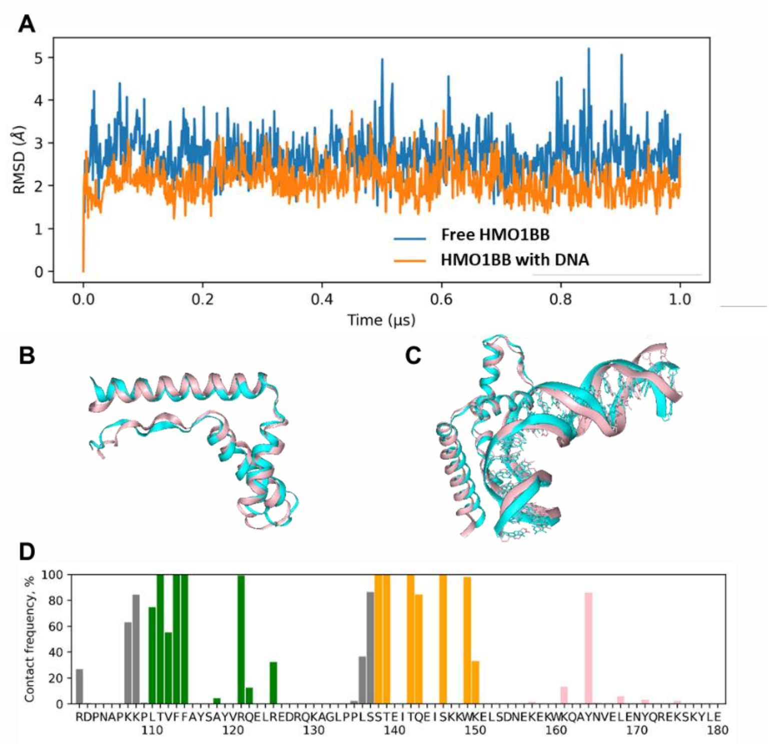

Figure S1. Modeling of the HMO1BB complex with DNA**.** (A) The RMSD fluctuations in the models of free HMO1BB and the protein-DNA complex during MD simulations. (B,C) Snapshots with superimposed initial and final structures of HMO1BB (B) and HMO1BB-DNA complex (C). (D) Frequency of HMO1BB contacts with DNA calculated from the MD trajectories. Residues of α-helices I, II and III are shown in green, yellow and pink, respectively. Residues of unordered regions of HMO1BB are shown in gray.
